# A Robust Manifold Graph Regularized Nonnegative Matrix Factorization Algorithm for Cancer Gene Clustering

**DOI:** 10.3390/molecules22122131

**Published:** 2017-12-02

**Authors:** Rong Zhu, Jin-Xing Liu, Yuan-Ke Zhang, Ying Guo

**Affiliations:** 1 School of Information Science and Engineering, Central South University, Changsha 410083, China; zhurongsd@126.com; 2 School of Information Science and Engineering, Qufu Normal University, Rizhao 276826, China; SDCAVELL@126.com (J.-X.L.); yuankezhang@163.com (Y.-K.Z.)

**Keywords:** robust, manifold, matrix factorization, gene clustering

## Abstract

Detecting genomes with similar expression patterns using clustering techniques plays an important role in gene expression data analysis. Non-negative matrix factorization (NMF) is an effective method for clustering the analysis of gene expression data. However, the NMF-based method is performed within the Euclidean space, and it is usually inappropriate for revealing the intrinsic geometric structure of data space. In order to overcome this shortcoming, Cai et al. proposed a novel algorithm, called graph regularized non-negative matrices factorization (GNMF). Motivated by the topological structure of the GNMF-based method, we propose improved graph regularized non-negative matrix factorization (GNMF) to facilitate the display of geometric structure of data space. Robust manifold non-negative matrix factorization (RM-GNMF) is designed for cancer gene clustering, leading to an enhancement of the GNMF-based algorithm in terms of robustness. We combine the $l_{2,1}$-norm NMF with spectral clustering to conduct the wide-ranging experiments on the three known datasets. Clustering results indicate that the proposed method outperforms the previous methods, which displays the latest application of the RM-GNMF-based method in cancer gene clustering.

## 1. Introduction

With the progressive implementation of human whole-genome and microarray technologies, it is possible to simultaneously observe the expressions of numerous genes in different tissue samples. By analyzing gene expression data, genes with varying expressions in tissues and their relationships may be identified to figure out the pathogenic mechanism of cancers based on genetic changes [1]. Recently, cancer classification based on gene expression data has become a hot research topic in bioinformatics.

Due to the fact that the analysis of genome-wide expression patterns can provide unique perspectives into the structure of genetic networks, the clustering technique has been used to analyze gene expression data [2,3]. Cluster analysis is the most widespread statistical techniques for analyzing massive gene expression data. Its major task is to classify genes with similar expressions to discover groups of genes with identical features or similar biological functions, in order that people can acquire a deeper understanding about the essence of many biological phenomena such as gene functions, development, cancer, and pharmacology [4].

Currently, it has been shown that non-negative matrix factorization (NMF) [5,6] is superior to the hierarchical clustering (HC) and self-organizing map (SOM) [7] in the application of cancer samples in clustering gene expression data. Over the past few years, the NMF-based method has been used for the gene expressions of statistically analyzing data for clustering [8,9,10,11,12,13]. The main idea is to approximately factorize a non-negative data matrix into a product of two non-negative matrices, which makes sure that all elements of the matrices are non-negative. Therefore, the appearance of NMF has attracted considerable attention. Recently, various variants based on the original NMF have been developed by modifying the objective function or the constraint conditions [14,15,16]. For instance, Cai et al. proposed graph regularized non-negative matrix factorization (GNMF), giving forth to the neighboring geometric structure. It illustrates the nearest neighbor graph that preserves the neighborhood information of high-dimensional space in low-dimensional space. The GNMF reveals the intrinsic geometrical structure by incorporating a Laplacian regularization term [17], which is effective for solving clustering problems. After that, the sparse NMF [18] was proposed with sparse constraints upon the basis matrices and coefficient matrices factored by the NMF so that the sparseness may be reflected from data. The non-smooth NMF [19] can realize the global or local sparseness [20] by making basis and encoding matrices sparse simultaneously. For the sake of enhancing the robustness of the GNMF-based method in gene clustering, we propose improved robust manifold non-negative matrix factorization (RM-GNMF) by making use of the combination of $l_{2,1}$-norm and spectral clustering with Laplacian regularization, leading to the internal geometry of data representations. It facilitates the display purposes of intrinsic geometric structure of the cancer gene data space.

This paper is organized as follows. In Section 2, we give a brief review on the NMF and the GNMF. In Section 3, we propose an improved RM-GNMF algorithm. In Section 4, we give the experimental results comparing with the previous methods. Finally, the conclusions are drawn in Section 5.

## 2. The NMF-Based and GNMF-Based Method

The NMF-based method [5] is a linear and non-negative approximate data description for non-negative matrices. We consider an original decomposed matrix *X* of size m×n, where *m* represents data characteristics and *n* represents the number of samples. Based on the NMF method, the matrix *X* is decomposed into two non-negative matrices $W\in R^{m\times r}$ and $H\in R^{n\times r}$, i.e.,

(1) $$X=WH^{T},$$

where r≤min(m,n). For a given decomposition $X=WH^{T}$ , sample *m* can be divided into *r* classes according to matrix $H^{T}$ . Each sample is placed in the highest metagene expression level in the sample, meaning that if $H_{ij}^{T}$ is the largest in column *j*, then sample *j* is assigned to class *i* .

Using the square of the Euclidean distance between *X* and $WH^{T}$ [21], we have the objective function of the NMF method

(2) $$O^{NMF}=||X-WH^{T}{\left|\right|}^{2}=\sum_{ij}(x_{ij}-\sum_{k=1}^{r}w_{ik}h_{jk}{)}^{2}.$$

According to the iterative update algorithms [6], the NMF-based method is performed on the basis of multiplicative update rules of *W* and *H* given by

(3) $$w_{ik}\leftarrow w_{ik}\frac{{\left(XH\right)}_{ik}}{{\left(WH^{T}H\right)}_{ik}},\;h_{jk}\leftarrow h_{jk}\frac{{\left(X^{T}W\right)}_{jk}}{{\left(HW^{T}W\right)}_{jk}}.$$

In order to overcome the limitation of the NMF-based method, Cai et al. [17] proposed the GNMF-based method, in which an affinity graph is generated to encode the geometrical information followed by a matrix factorization with respect to the graph structure. In contrast to the NMF-based method, it has a regular graph constraint, which preserves the advantage of the local sparse representation of the NMF-based method and preserves the similarity between the original data points after dimensionality reduction.

There are several weighting schemes, such as zero-one weighting, heat kernel weighting, and Gaussian weighting [17]. In what follows, we consider the zero-one weighting described as

(4) $$Q_{ij}=\left\{\begin{array}{cc} 1, & \mathrm{if}\;x_{i}\in N_{k}\left(x_{j}\right)\;\mathrm{or}\;x_{j}\in N_{k}\left(x_{i}\right),\\ 0, & \mathrm{otherwise}. \end{array}\right.$$

Based on the weight matrix *Q*, we obtain the objective function of the GNMF method given by

(5) $$O^{GNMF}=||X-WH^{T}{\left|\right|}^{2}+\lambda Tr\left(H^{T}LH\right),$$

where Tr(·) denotes the trace of matrices and L=D−Q. *D* is a diagonal matrix whose entries are column or row sums of *Q* with $D_{jj}=\sum_{l}Q_{jl}$ [22]. The regularization parameter λ≥0 can be used for the smoothness control of the new representation. By the iterative algorithms to minimize the objective function $O^{GNMF}$ , we achieve the updating rules

(6) $$w_{ik}\leftarrow w_{ik}\frac{{\left(XH\right)}_{ik}}{{\left(WH^{T}H\right)}_{ik}},\;h_{jk}\leftarrow h_{jk}\frac{{\left(X^{T}W+\lambda WH\right)}_{jk}}{{\left(HW^{T}W+\lambda DH\right)}_{jk}}.$$

## 3. The RM-GNMF-Based Method for Gene Clustering

So far, we have described the NMF-based method and GNMF-based method. In what follows, we seek RM-GNMF gene clustering by making use of the combination of $l_{2,1}$-norm and spectral clustering with the Laplacian regularization.

### 3.1. The $l_{2,1}$-Norm

The $l_{2,1}$-norm of a matrix was initially employed as a rotational invariant $l_{1}$-norm [23], which was usually used for multi-task learning [24,25] and tensor factorization [26]. Instead of using $l_{2}$-norm-based loss function that is sensitive to outliers, we resort to the $l_{2,1}$-norm-based loss function and regularization [23], which is convergence-proved.

For the sake of getting over the drawbacks of the NMF-based method and enhancing the robustness of the GNMF-based method, we employ the $l_{2,1}$-norm for the matrix factorization in the RM-GNMF-based method. For a non-negative matrix *X* of size m×n, the $l_{2,1}$-norm of matrice *X* is defined as

(7) $${\left||X|\right|}_{2,1}=\sum_{i=1}^{n}\left|\right|x_{i}{\left|\right|}_{2},$$

where data vectors are arranged in columns, and the $l_{2,1}$-norm calculates the $l_{2}$-norm for column vectors first. Subsequently, the matrix factorization assignment becomes

(8) $$\min_{H\geq 0}||X-WH^{T}{\left|\right|}_{2,1}.$$

### 3.2. Spectral-Based Manifold Learning to Constrained GNMF

The spectral method is a classical method of analysis and algebra in mathematics. It is widely used in low dimensional representation and clustering problems of high dimensional data [27,28]. A relational matrix describing the similarity of the pair of data points is defined according to the given sample dataset, and the eigenvalues and eigenvectors of the matrices are calculated. After that, the appropriate eigenvectors are selected and the low dimensional embedding of the data is obtained. The degree matrices are defined on a given graph, such as an adjacency matrix of the graph, a Laplacian matrix, and so on [22].

Based on the spectrum of the matrices with respect to the graph, spectral theory further reveals the information contained in the graph , and establishes the connection between the discrete space and the continuous space through the techniques of geometry, analysis, and algebra. It has a wide range of applications in manifold learning. In this section, the *p*-nearest neighbor method can be used for establishing the relationship between each data point and its neighborhood.

For a data matrix $X\in R^{m\times n}$, we treat each column of *X* as a data point and each data point as a vertex, respectively. The *p*-nearest-neighbor graph *G* can be constructed with *n* vertices. Then the symmetric weight matrix $Q\in R^{n\times m}$ is generated, where the element $q_{ij}$ denotes the weight of the edge joining vertices *i* and *j* and the value of $q_{ij}$ is given by

(9) $$q_{ij}=\left\{\begin{array}{cc} 1, & \mathrm{if}\;x_{i}\in N_{p}\left(x_{j}\right)\;\mathrm{or}\;d_{j}\in N_{p}\left(x_{i}\right),\\ 0, & \mathrm{otherwise}. \end{array}\right.$$

where $N_{p}\left(x_{i}\right)$ denotes the set of *p*-nearest neighbors of $x_{i}$. It is obvious that the matrix *Q* represents the affinity between the data points.

There is an assumption about manifold. Namely, if two data points $x_{i}$ and $x_{j}$ are close in the intrinsic geometric structure of the data distribution, then their presentations under a new basis will be close [29]. Therefore, we define the relationship as follows

(10) $$\min_{x_{p}}\sum_{ij}\left|\right|x_{i}-x_{j}{\left|\right|}^{2}q_{ij},$$

where $m_{i}$ and $m_{j}$ denote the mappings of $x_{i}$ and $x_{j}$, respectively. The degree matrix *D* is a diagonal matrix given by $d_{ii}=\sum_{j}q_{ij}$. Obviously, $d_{ii}$ is the sum of all the similarities related with $x_{i}$. Then, the graph Laplacian matrix is given by

(11) L=D−Q.

The graph embedding can be written as

(12) $$\begin{array}{ccc} \min_{x}\sum_{ij}\left|\right|x_{i}-x_{j}{\left|\right|}^{2}q_{ij} & = & \min_{X}tr\left(X\left(D-Q\right)X^{T}\right)\\ & = & \min_{X}tr\left(XLX^{T}\right). \end{array}$$

In the RM-GNMF-based method, we combine the GNMF-based method with the spectral clustering, resulting in the $l_{2,1}$-norm constrained GNMF as follows

(13) $$O^{RMGNMF}=||X-WH^{T}{\left|\right|}_{2,1}+\lambda Tr\left(H^{T}LH\right),$$

where λ≥0 is the regularization parameter. We resort to the augmented Lagrange multiplier (ALM) method to solve the above problem.

For an auxiliary variable $Z=X-WH^{T}$, we rewrite the $O^{RMGNMF}$ in Equation (13) as

(14) $$\min_{W,H,Z}{\left||Z|\right|}_{2,1}+\alpha Tr\left(H^{T}LH\right),$$

satisfying the constraints $Z-X+WH^{T}=0$ and $H^{T}H=I$. Then, we define the augmented Lagrangian function

(15) $$\begin{array}{ccc} L_{\mu }\left(Z,W,H,\Lambda \right)\;\;\;\; & = & {\;\;\;\;||Z||}_{2,1}+Tr\Lambda^{T}\left(Z-X+WH^{T}\right)+\frac{\mu }{2}||Z-X+WH^{T}{\left|\right|}_{2,1}\\ & & \;+\alpha Tr(H^{T}LH). \end{array}$$

satisfying the constraint $H^{T}H=I$, where μ is the step size of update and Λ is the Lagrangian multiplier.

To optimize Equation (15), we rewrite the objective function to get the following task

(16) $$L_{\mu }\left(Z,W,H,\Lambda \right)={\left||Z|\right|}_{2,1}+\frac{\mu }{2}||Z-X+WH^{T}+\frac{\Lambda }{\mu }{\left|\right|}_{F}^{2}+\alpha Tr\left(H^{T}LH\right),$$

satisfying the constraint $H^{T}H=I$.

### 3.3. Computation of *Z*

For the given *W* and *H*, we can solve *Z* in Equation (15) by using the update of *Z* related to the following issue

(17) $$Z^{r+1}=arg\min_{Z}{\left||Z|\right|}_{2,1}+\frac{\mu }{2}||Z-(X-W^{r}{\left(H^{r}\right)}^{T}-\frac{\Lambda^{r}}{\mu }{\left|\right|}_{F}^{2}.$$

We need the following Lemma to solve *Z* in Equation (15). Please see the Appendix for a detailed proof.

**Lemma** **1.** Given a matrix $W=\left[w_{1},\cdots ,w_{n}\right]\in R^{m\times n}$ and a positive scalar λ, $Z^{*}$ is the optimal solution of

(18) $$\min\frac{1}{2}||Z-{W||}_{F}^{2}+{\lambda ||Z||}_{2,1},$$

and the i-th column of $Z^{*}$ is given by

(19) $$Z^{*}\left(:,i\right)=\left\{\begin{array}{cc} \frac{\left|\right|w_{i}\left|\right|-\lambda }{\left|\right|w_{i}\left|\right|}W_{i},\; & if\;\lambda <||W_{i}\left|\right|,\\ 0, & \mathrm{otherwise}. \end{array}\right.$$

### 3.4. Computation of W and H

For the given other parameters, we solve the optimal *W*. The update of *W* amounts to solve

(20) $$W^{r+1}=\arg\;\min_{W}\frac{\mu }{2}\left|\right|Z^{r}-X+W^{r}{\left(H^{r}\right)}^{T}+\frac{\Lambda }{\mu }{\left|\right|}_{F}^{2}.$$

Let $X-Z+\frac{\Lambda }{\mu }=M$. The problem in Equation (20) can be rewritten as

(21) $$W^{r+1}=\arg\;\min_{W}\frac{\mu }{2}||M+W^{r}{\left(H^{r}\right)}^{T}{\left|\right|}_{F}^{2}.$$

If the partial derivative of *W* is set to be 0, we obtain

(22) $$W^{r+1}=MH^{r}.$$

Then, we derive the optimal *H*. Taking W=MH, the update problem of *H* can be expressed as

(23) $$H^{r+1}=\arg\min_{H}\frac{\mu }{2}||M-MHH^{T}{\left|\right|}_{F}^{2}+\alpha Tr\left(H^{T}LH\right),$$

satisfying the constraint $H^{T}H=I$. We have

(24) $$\begin{array}{ccc} H^{r+1} & = & \arg\min_{H}||M-MHH^{T}{\left|\right|}_{F}^{2}+\frac{2\alpha }{\mu }Tr\left(H^{T}LH\right)\\ & = & arg\min_{H}TrH^{T}\left(-M^{T}M+\frac{2\alpha }{\mu }L\right)H. \end{array}$$

Therefore, the optimal $H^{r+1}$ can be achieved by counting eigenvectors of

(25) $$H^{r+1}=\left(h_{1},\cdots ,h_{k}\right).$$

### 3.5. Updating of Λ and μ

The update standard of Λ and μ can be described as follows

(26) $$\begin{array}{cc} & \Lambda^{r+1}=\Lambda^{r}+\mu \left(Z^{r+1}-X+W^{r}{\left(H^{r}\right)}^{T}\right), \end{array}$$

(27) $$\begin{array}{c} \mu^{r+1}=p\mu^{r}, \end{array}$$

where *p* is the nearest neighbor graph parameter. The detailed process of the RM-GNMF-based method is listed in Algorithm 1. 

**Algorithm 1:** The RM-GNMF-based Algorithm **Input**: The dataset $X=\left[x_{1},x_{2},\cdots ,x_{n}\right]\in R^{m\times n}$, a predefined number of clusters *k*, parameters μ, λ, the nearest neighbor graph parameter *p*, maximum iteration number $t_{max}$. **Initialization**:  Z=Λ=0, $W_{0}\in R^{m\times k}$, $H_{0}\in R^{k\times n}$. **Repeat** Fix other parameters, and then update *Z* by formula (17); Fix other parameters, and then update *W* by: $W=(X-Z+\frac{\Lambda }{\mu })H$; Update *H* by $H=UV^{T}$, where *U* and *V* are the left and right singular values of the SVD decomposition; Fix other parameters, and then update Λ and μ by formulas (26)(27); t = t + 1; Until $t\geq t_{max}$.  **Output**: matrix $W\in R^{m\times k}$ , matrix $H\in R^{k\times n}$.


## 4. Results and Discussion

In this section, we evaluate the performance of the proposed method on the three gene expression datasets. We compare the RM-GNMF-based method with the NMF-based method [6], the $l_{2,1}$-NMF-based method [23], the LNMF-based method [20], and the GNMF-based method [17].

### 4.1. Datasets

In order to evaluate the performance of proposed RM-GNMF algorithm, the clustering experiment was conducted on several gene expressions datasets of cancer patients. Three classical genetic datasets were used in the experiment, including leukemia [1], colon, and GLI_85 [30]. These gene expression datasets are downloaded from: http://featureselection.asu.edu/datasets.php.The colon cancer datasets consist of the gene expression profiles of 2000 genes for 62 tissue samples among which 40 are colon cancer tissues and 22 are normal tissues. The leukemia datasets consist of 7129 genes and 72 samples (47 ALL and 25 AML).

A brief description of experimental datasets is described in Table 1.

More detailed information on these datasets can be found in the relevant references, and these datasets are available for download from the reference website.

### 4.2. Evaluation Metrics

For the sake of evaluating the clustering results, we use the clustering accuracy and normalized mutual information (NMI) to demonstrate the performance of the proposed algorithm.

Clustering accuracy can be calculated as

(28) $$ACC=\frac{\sum_{i=1}^{n}\delta \left(map\left(c_{i}\right),l_{i}\right)}{n},$$

where $c_{i}$ is the cluster label of $x_{i}$, and $l_{i}$ is the true class label *i*-th sample, *n* denotes the total number of samples, and $\delta (map\left(c_{i}\right),l_{i})$ is a delta function. If $map\left(c_{i}\right)=l_{i}$, we obtain $\delta (map\left(c_{i}\right),l_{i})=1$, where $map(c_{i})$ is the mapping function that maps the cluster label $c_{i}$ into the actual label $l_{i}$. Otherwise, we have $\delta (map\left(c_{i}\right),l_{i})=0$. We can find the best mapping by the Kuhn–Munkres method [31]. NMI can be described as

(29) $$NMI=\frac{\sum_{i=1}^{N}\sum_{j=1}^{N}n_{i,j}log\frac{n_{i,j}}{n_{i}{\hat{n}}_{j}}}{\sqrt{\left(\sum_{i=1}^{N}n_{i}log\frac{n_{i}}{n}\right)\left(\sum_{j=1}^{N}{\hat{n}}_{j}log\frac{{\hat{n}}_{j}}{n}\right)}},$$

where $n_{i}$ is the size of the *i*-th cluster and ${\hat{n}}_{j}$ is the size of the *j*-th class, $n_{i,j}$ is the number of data between the intersections, and *N* denotes the number of clusters. We perform 100 experiments under each target feature dimension, taking the mean of the accurate and NMI values as the experimental results.

### 4.3. Parameter Selection

The RM-GNMF-based method involves two essential parameters, i.e., the regularization parameter λ and the regularity coefficient μ determining the penalty for infeasibility.

**Figure 1:**
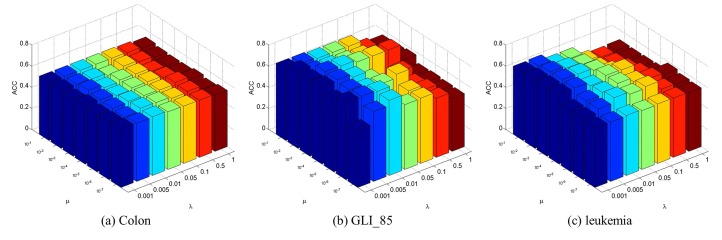
Influence of parameters on clustering accuracy.

We set the parameters λ and μ in the range of λ∈{0.001,0.005,0.01,0.05,0.1,0.5,1} and $\mu \in \{10^{-1},10^{-2},10^{-3},10^{-4},10^{-5},10^{-6},10^{-7}\}$ . We use the cross-validation method to get the best parameter values λ=0.05 and $\mu =10^{-3}$. In order to intuitively to analyze the influence of parameters λ and μ of the RM-GNMF-based method on the accuracy of clustering, Figure 1 shows the variation on clustering accuracy when the two parameters are modified. The three subgraphs in Figure 1 correspond to three gene expression datasets respectively. As can be viewed in Figure 1, the parameter $\mu =10^{-3}$ can get higher ACC. With the change of regular parameter λ, the change of ACC is relatively flat, and the clustering accuracy is higher when the value of λ is smaller. Therefore, we set $\lambda =0.05,\mu =10^{-3}$ in the follow-up experiments.

### 4.4. Clustering Results

In Table 2, we demonstrate the clustering results on the colon, GLI_85 and leukemia datasets, respectively. Reported is the mean of clustering results from 100 runs of different NMF methods together.

It can be found that the RM-GNMF-based method outperforms the original NMF-based method, while the RM-GNMF-based method achieves the best performance compared with the other three datasets. The clustering accuracies of the RM-GNMF-based method are 66.13%,75.29%, and 65.28% for the colon, GLI_85, and leukemia datasets, respectively.

Our tests on several gene expression profiling datasets of cancer patients consistently indicate that the RM-GNMF-based method achieves significant improvements in comparison with the NMF-based method, the $l_{2,1}$-NMF-based method, the LNMF-based method, and the GNMF-based method, in terms of cancer prediction accuracy.

**Figure 2:**
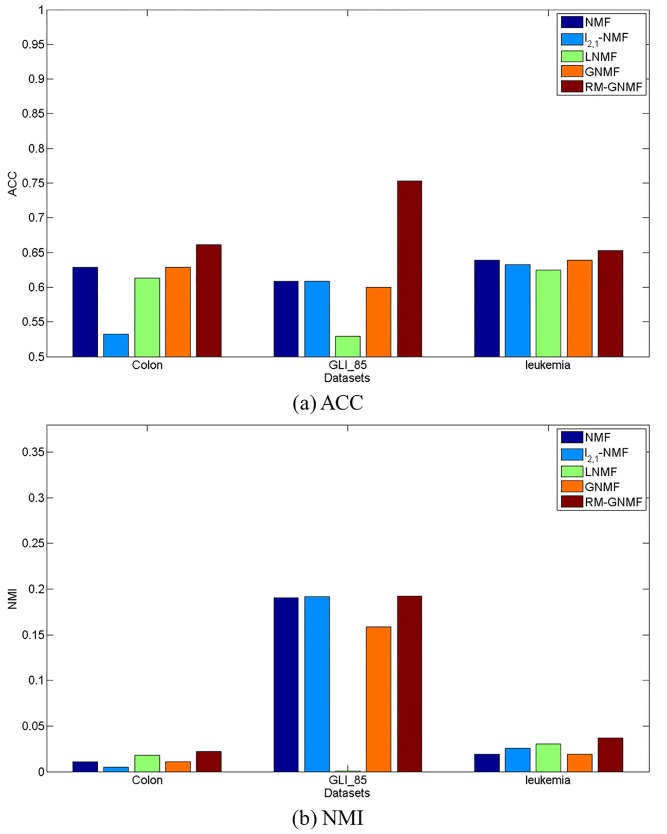
Clustering results on different datasets.

As shown in Figure 2, the RM-GNMF-based method always gives birth to better clustering results than other NMF-based method using the three original datasets.

To demonstrate the robustness of our approach to data changes, we add uniform noise onto the three gene expression datasets. A disturbed matrix $Y_{noise}$ is generated by adding independent uniform noise, defined as follows:

(30) $$Y_{noise}=Y+r,$$

where *Y* is the original matrix, *r* is a random number generated by a uniform distribution on the interval [0,max], and max is the maximum expression of *Y* .

**Figure 3:**
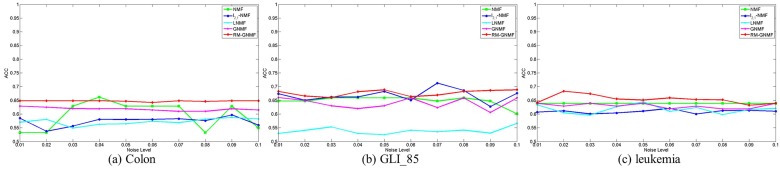
Influence of noise on clustering accuracy.

The experimental results with noise added are shown in Figure 3. It can be seen that the clustering result of RM-GNMF algorithm is still stable with the addition of noise, which shows that RM-GNMF algorithm is robust.

In order to verify the results obtained from the algorithms in the experiments, we import the clustering result of the comparison methods into STAC web platform to perform the statistical test (http://tec.citius.usc.es/stac/). We selected the Friedman test of non-parametric multiple groups; the significance level is 0.05. The analysis results obtained are presented in Table 3 and Table 4.

From the above test results it can be concluded that H0 is rejected. Hence, we believe that the clustering results of five algorithms are significantly different.

## 5. Conclusions

We have proposed the RM-GNMF-based method with the $l_{2,1}$-norm and spectral-based manifold learning. This algorithm is suitable for cancer gene expression data clustering with an elegant geometric structure. Our tests on several gene expression profiling datasets of cancer patients consistently indicate that the RM-GNMF-based method achieves significant improvements in comparison with the NMF-based method, the $l_{2,1}$-NMF-based method, the LNMF-based method, and the GNMF-based method, in terms of cancer prediction accuracy and robustness.

## Figures and Tables

**Table 1:** Statistics of three gene expression datasets.

Data Sets	Instances	Features	Classes
Colon	62	2000	2
GLI_85	85	22,283	2
Leukemia	72	7070	2

**Table 2:** Clustering results on different datasets. NMF: non-negative matrix factorization; GNMF: graph regularized non-negative matrices factorization; RM-GNMF: robust manifold non-negative matrix factorization; NMI: normalized mutual information.

Methods	Colon		GLI_85		Leukemia	
	ACC	NMI	ACC	NMI	ACC	NMI
NMF	0.6290	0.0110	0.6088	0.1906	0.6389	0.0193
$L_{2,1}$-NMF	0.5323	0.0048	0.6088	0.1916	0.6328	0.0258
LNMF	0.6129	0.0181	0.5294	0.0011	0.6250	0.0306
GNMF	0.6290	0.0110	0.6000	0.1584	0.6389	0.0193
RM-GNMF	0.6613	0.0220	0.7529	0.1925	0.6528	0.0369

**Table 3:** Friedman test (significance level of 0.05).

Statistic	*p*-Value	Result
7.00000	0.01003	H0 is rejected

**Table 4:** Ranking.

Rank	Algorithm
1.33333	LNMF
2.33333	NMF
2.66667	$L_{2,1}$-NMF
3.66667	GNMF
5.00000	RM-GNMF

## Appendix A

**Lemma** **A1.** *With the given matrix $W=\left[w_{1},\cdots ,w_{n}\right]\in R^{m\times n}$ and the positive scalar λ, $Z^{*}$ is the optimal solution of*

(A1) $$\min\frac{1}{2}||Z-{W||}_{F}^{2}+{\lambda ||Z||}_{2,1},$$

*and the i-th column of $Z^{*}$ can be calculated as*

(A2) $$Z^{*}\left(:,i\right)=\left\{\begin{array}{cc} \frac{\left|\right|w_{i}\left|\right|-\lambda }{\left|\right|w_{i}\left|\right|}W_{i},\; & if\;\;\lambda <||W_{i}\left|\right|,\\ 0, & \mathrm{otherwise}. \end{array}\right.$$

**Proof.** The objection function in Equation (A1) is equivalent to the following equation

(A3) $$\sum_{i=1}^{n}\left|\right|z_{i}-w_{i}{\left|\right|}_{F}^{2}+\lambda \sum_{i=1}^{n}\left|\right|z_{i}{\left|\right|}_{2},$$

which can be solved in a decoupled manner

(A4) $$\min_{z_{i}}\frac{1}{2}\left|\right|z_{i}-w_{i}{\left|\right|}_{2}^{2}+\lambda ||z_{i}{\left|\right|}_{2}.$$

After taking derivative with respect to $z_{i}$, we get

(A5) $$\frac{\partial ||z_{i}{\left|\right|}_{2}}{\partial z_{i}}=\left\{\begin{array}{cc} r, & \mathrm{if}\;z_{I}=0\\ \frac{z_{i}}{\sqrt{z_{i}^{T}z_{i}}}, & \mathrm{otherwise}, \end{array}\right.$$

where *r* is a subgradient vector and ${\left||r|\right|}_{2}\leq 1$. For $z_{i}=0$, we get

(A6) $$-w_{i}+\lambda r=0,$$

where $\lambda \geq \left|\right|w_{i}\left|\right|$. For $z_{i}\neq 0$, we get

(A7) $$z_{i}-w_{i}+\lambda \frac{z_{i}}{\sqrt{z_{i}^{T}z_{i}}}=0.$$

Combining Equation (A6) with Equation (A7), we obtain

(A8) $$z_{i}=\alpha w_{i},$$

where $\alpha =\frac{\left|\right|z_{i}{\left|\right|}_{2}}{\left|\right|z_{i}{\left|\right|}_{2}+\lambda }>0$. Plugging $z_{i}$ in Equation (A8) to Equation (A7), we solve α, which is substituted back into Equation (A8). After performing the above-mentioned steps, we obtain

(A9) $$z_{i}=\left(1-\frac{\lambda }{\left|\right|w_{i}\left|\right|}\right)w_{i}.$$

 □
